# Supplementary material for: Rev7 and 53BP1/Crb2 prevent RecQ helicase-dependent hyper-resection of DNA double-strand breaks
Source: eLife. 2018 Apr 26;7:e33402. doi: 10.7554/eLife.33402 (PMC5945276; doi:10.7554/eLife.33402)
Supplement: Supplementary file 1. — These strains were for all LacO resection assay experiments in Figures 1–3 unless otherwise noted. PDis1-GFP-LacI-NLS is derived from Shimada et al. (2003). LacO integrations were performed as described in Leland and King, 2014. Strains containing the RMCE Purg1lox expression system were derived from Watson et al. (2011). [file elife-33402-supp1.docx]

Table 1

| **Strain** | **Description** | **Complete Genotype** |
| --- | --- | --- |
| MKSP1381 | Original LacO integration used to generate all other strains in this study (Figure S1B) | *h- leu1-32 ura4-D18 his7+:P_Dis1_-GFP-LacI-NLS ChrII:3442981::Ura4-10.3kbLacO ChrII:3446249::HOcs-hphMX6 Cut11-mCherry::natMX6* |
| MKSP1173 | Example of LacO array contracting upon integration (Figure S1B) | *h- leu1-32 ura4-D18 his7+:P_Dis1_-GFP-LacI-NLS Leu2::Ura4-LacO<5kb Rad26-mCherry::natMX6* |
| MKSP2123 | WT LacO resection assay | *h- leu1-32 ura4-D18 his7+:P_Dis1_-GFP-LacI-NLS ChrII:3442981::Ura4-10.3kbLacO ChrII:3446249::HOcs-bleMX6 Rad52-mCherry::bleMX6 urg1::RMCE-bleMX6* |
| MKSP1914 | *exo1*∆ LacO resection assay | *h- leu1-32 ura4-D18 his7+:P_Dis1_-GFP-LacI-NLS ChrII:3442981::Ura4-10.3kbLacO ChrII:3446249::HOcs-hphMX6 Rad52-mCherry::natMX6 urg1::RMCE-kanMX6 exo1::bleMX6* |
| MKSP2501 | *rqh1*∆ LacO resection assay | *h- leu1-32 ura4-D18 his7+:P_Dis1_-GFP-LacI-NLS ChrII:3442981::Ura4-10.3kbLacO ChrII:3446249::HOcs-hphMX6 Rad52-mCherry::bleMX6 urg1::RMCE-bleMX6 rqh1::kanMX6* |
| MKSP2244 | *crb2*∆ LacO resection assay | *h- leu1-32 ura4-D18 his7+:P_Dis1_-GFP-LacI-NLS ChrII:3442981::Ura4-10.3kbLacO ChrII:3446249::HOcs-hphMX6 Rad52-mCherry::bleMX6 urg1::RMCE-bleMX6 crb2::kanMX6* |
| MKSP2476 | *crb2*∆*exo1*∆ LacO resection assay | *h+ leu1-32 ura4-D18 his7+:P_Dis1_-GFP-LacI-NLS ChrII:3442981::Ura4-10.3kbLacO ChrII:3446249::HOcs-hphMX6 Rad52-mCherry::bleMX6 urg1::RMCE-bleMX6 crb2::kanMX6 exo1::bleMX6* |
| MKSP2479 | *crb2*∆*rqh1*∆ LacO resection assay | *h+ leu1-32 ura4-D18 his7+:P_Dis1_-GFP-LacI-NLS ChrII:3442981::Ura4-10.3kbLacO ChrII:3446249::HOcs-hphMX6 Rad52-mCherry::bleMX6 urg1::RMCE-bleMX6 crb2::kanMX6 rqh1::kanMX6* |
| MKSP2149 | *rev7*∆ LacO resection assay | *h+ leu1-32 ura4-D18 his7+:P_Dis1_-GFP-LacI-NLS ChrII:3442981::Ura4-10.3kbLacO ChrII:3446249::HOcs-hphMX6 Rad52-mCherry::bleMX6 urg1::RMCE-bleMX6 rev7::natMX6* |
| MKSP2392 | *rev7*∆*exo1*∆ LacO resection assay | *h+ leu1-32 ura4-D18 his7+:P_Dis1_-GFP-LacI-NLS ChrII:3442981::Ura4-10.3kbLacO ChrII:3446249::HOcs-hphMX6 Rad52-mCherry::bleMX6 urg1::RMCE-bleMX6 rev7::natMX6 exo1::bleMX6* |
| MKSP2456 | *rev7*∆*rqh1*∆ LacO resection assay | *h+ leu1-32 ura4-D18 his7+:P_Dis1_-GFP-LacI-NLS ChrII:3442981::Ura4-10.3kbLacO ChrII:3446249::HOcs-hphMX6 Rad52-mCherry::bleMX6 urg1::RMCE-bleMX6 rev7::natMX6 rqh1::kanMX6* |
| MKSP2245 | *rev7*∆*crb2*∆ LacO resection assay | *h+ leu1-32 ura4-D18 his7+:P_Dis1_-GFP-LacI-NLS ChrII:3442981::Ura4-10.3kbLacO ChrII:3446249::HOcs-hphMX6 Rad52-mCherry::bleMX6 urg1::RMCE-bleMX6 rev7::natMX6 crb2::kanMX6* |
| MKSP2262 | *rev3*∆ LacO resection assay | *h+ leu1-32 ura4-D18 his7+:P_Dis1_-GFP-LacI-NLS ChrII:3442981::Ura4-10.3kbLacO ChrII:3446249::HOcs-hphMX6 Rad52-mCherry::bleMX6 urg1::RMCE-bleMX6 rev3::kanMX6* |
| MKSP2477 | *ctp1*∆ LacO resection assay | *h+ leu1-32 ura4-D18 his7+:P_Dis1_-GFP-LacI-NLS ChrII:3442981::Ura4-10.3kbLacO ChrII:3446249::HOcs-hphMX6 Rad52-mCherry::bleMX6 urg1::RMCE-bleMX6 ctp1::kanMX6* |
