# Supplementary material for: Rev7 and 53BP1/Crb2 prevent RecQ helicase-dependent hyper-resection of DNA double-strand breaks
Source: eLife. 2018 Apr 26;7:e33402. doi: 10.7554/eLife.33402 (PMC5945276; doi:10.7554/eLife.33402)
Supplement: Supplementary file 2. — These counts pertain to all data from the resection assay in Figures 1–3. [file elife-33402-supp2.docx]

Table 2

| **Genotype** | **Number of biological replicates** | **Total cell cycles observed** | **Number of dead or sick cells*** | **Number of daughter cell cycles whose parent had an on-target DSB*** |  | **Observed cell cycles valid for  %Rad52 calculations** | **Number of Rad52 focus formation events** | **Number of resection events that are exactly determined** |
| --- | --- | --- | --- | --- | --- | --- | --- | --- |
| WT | 6 | 1049 | 27 | 52 |  | 486 | 77 | 45 |
| *exo1*∆ | 4 | 945 | 13 | 2 |  | 508 | 8 | 0 |
| *rqh1*∆ | 6 | 991 | 61 | 2 |  | 556 | 49 | 32 |
| *crb2*∆ | 5 | 1045 | 78 | 164 |  | 425 | 62 | 48 |
| *crb2*∆*exo1*∆ | 4 | 1122 | 42 | 128 |  | 579 | 64 | 44 |
| *crb2*∆*rqh1*∆ | 3 | 1207 | 73 | 70 |  | 714 | 83 | 27 |
| *rev7*∆ | 5 | 3182 | 190 | 24 |  | 1851 | 112 | 54 |
| *rev7*∆*exo1*∆ | 8 | 1251 | 70 | 2 |  | 702 | 26 | 11 |
| *rev7*∆*rqh1*∆ | 7 | 1311 | 57 | 10 |  | 809 | 130 | 50 |
| *rev7*∆*crb2*∆ | 3 | 1122 | 43 | 88 |  | 477 | 65 | 42 |
| *rev3*∆ | 2 | 637 | 21 | 24 |  | 275 | 43 | 31 |

* Cell cycles that were excluded from the analyses of resection rate in Figures 1F, 2C, 3A.
