# Supplementary material for: Rev7 and 53BP1/Crb2 prevent RecQ helicase-dependent hyper-resection of DNA double-strand breaks
Source: eLife. 2018 Apr 26;7:e33402. doi: 10.7554/eLife.33402 (PMC5945276; doi:10.7554/eLife.33402)
Supplement: Supplementary file 3. [file elife-33402-supp3.docx]

Table 3

| ApoI site position relative to HOcs at Mmf1 | Forward primer (5’-3’) | Reverse primer (5’-3’) |
| --- | --- | --- |
| -168v1 | ATCACCGATGGAAACAGTGAACTCAT | GCTGATTTGCTAGCAGTCTTCAGCTC |
| -168v2 | GGAAACAGTGAACTCATATCATATCCA | GCAGTCTTCAGCTCAGATAATAAGG |
| -300 | AGAAACTTTTACAAACCTCGCGT | TGAGTTCACTGTTTCCATCGGT |
| -3023 | AGCTTGTAATAATCGATGCCAAAGG | GTTGAGGCTAAACGACCCATT |
| +13150 | GCCAGCTATGACAAAAGGCC | TAGGATCGTAGTTGCCAGCG |
| -14253 | AGCTGGTTGGAAGGCATATCA | CGCAAACAAGGCATCGACTTT |
| Control primers |  |  |
| Ncb1v0 | AGACGTATTTGAGTGATAGTGCTCGCTGC | CGTCCTTCCGATGTTGCTTTAACGCATACTC |
| Ncb1v1 | GCCGCTGAACACATTATTAAAGC | CGCCACTTCCAAAGCTTCAG |
| spanning HOcs@Mmf1 | GGAGGCCCAGAATACAGTTTC | CGCACGTCAAGACTGTCAAG |

The -168v1 and Ncb1v0 primers were used in panel Figure 1 – figure supplement 3C. For all other plots, the -168v2 and Ncb1v1 primers were used.
