# Supplementary material for: Rev7 and 53BP1/Crb2 prevent RecQ helicase-dependent hyper-resection of DNA double-strand breaks
Source: eLife. 2018 Apr 26;7:e33402. doi: 10.7554/eLife.33402 (PMC5945276; doi:10.7554/eLife.33402)
Supplement: Transparent reporting form [file elife-33402-transrepform.docx]

***eLife’s* transparent reporting form**

We encourage authors to provide detailed information *within their submission* to facilitate the interpretation and replication of experiments. If you have any questions, please contact us: [editorial@elifesciences.org](mailto:editorial@elifesciences.org).

**Sample-size estimation**

- You should state whether an appropriate sample size was computed when the study was being designed
- You should state the statistical method of sample size computation and any required assumptions
- If no explicit power analysis was used, you should describe how you decided what sample (replicate) size (number) to use

Please outline where this information can be found within the submission (e.g., page numbers or figure legends), or explain why this information doesn’t apply to your submission:

We decided on a total number of fields to collect/analyze (400) first, and then randomized, blinded fields were analyzed until this number was reached before performing any statistical tests. We could not collect/analyze fields until a specific sample size was reached because the frequency of analyzable cell cycles was variable. For example, different genotypes had higher or lower percentages of cell cycles with on-target Rad52 loading events, different experimental runs had different degrees of photobleaching (photobleaching was controlled for first, as described in the Methods). Relevant information about sample size is in Supplementary File 2.

**Replicates**

- You should report how often each experiment was performed
- You should include a definition of biological versus technical replication
- The data obtained should be provided and sufficient information should be provided to indicate the number of independent biological and/or technical replicates
- If you encountered any outliers, you should describe how these were handled
- Criteria for exclusion/inclusion of data should be clearly stated
- High-throughput sequence data should be uploaded before submission, with a private link for reviewers provided (these are available from both GEO and ArrayExpress)

Please outline where this information can be found within the submission (e.g., page numbers or figure legends), or explain why this information doesn’t apply to your submission:

Information on replicates is provided in detail in Supplementary File 2. For each fission yeast genotype, this table includes the number of biological replicates, the number of individual cells imaged, the number of cells excluded based on the defined criteria (as described in the Methods), and finally the number of cells that were used to generate each plot in the Figures.

**Statistical reporting**

- Statistical analysis methods should be described and justified
- Raw data should be presented in figures whenever informative to do so (typically when N per group is less than 10)
- For each experiment, you should identify the statistical tests used, exact values of N, definitions of center, methods of multiple test correction, and dispersion and precision measures (e.g., mean, median, SD, SEM, confidence intervals; and, for the major substantive results, a measure of effect size (e.g., Pearson's r, Cohen's d)
- Report exact p-values wherever possible alongside the summary statistics and 95% confidence intervals. These should be reported for all key questions and not only when the p-value is less than 0.05.

The methods for statistical analysis are described in each Figure Legend, the sample sizes are given in Supplementary File 2, and the p-values are explicitly stated.

Please outline where this information can be found within the submission (e.g., page numbers or figure legends), or explain why this information doesn’t apply to your submission:

(For large datasets, or papers with a very large number of statistical tests, you may upload a single table file with tests, Ns, etc., with reference to page numbers in the manuscript.)

**Additional data files (“source data”)**

- We encourage you to upload relevant additional data files, such as numerical data that are represented as a graph in a figure, or as a summary table
- Where provided, these should be in the most useful format, and they can be uploaded as “Source data” files linked to a main figure or table
- Include model definition files including the full list of parameters used
- Include code used for data analysis (e.g., R, MatLab)
- Avoid stating that data files are “available upon request”

Please indicate the figures or tables for which source data files have been provided:

The complete datasets and code for the analysis pipeline are currently available on github (https://github.com/lelandbr/Leland_King_2018_eLife_Rev7_EndResection)
